# Subjective and Objective Quality Assessment of Swimming Pool Images

**DOI:** 10.3389/fnins.2021.766762

**Published:** 2022-01-11

**Authors:** Fei Lei, Shuhan Li, Shuangyi Xie, Jing Liu

**Affiliations:** Faculty of Information Technology, Beijing University of Technology, Beijing, China

**Keywords:** image quality assessment, subjective/objective quality assessment, swimming pool image database, main target extraction, multi-feature fusion

## Abstract

As the research basis of image processing and computer vision research, image quality evaluation (IQA) has been widely used in different visual task fields. As far as we know, limited efforts have been made to date to gather swimming pool image databases and benchmark reliable objective quality models, so far. To filled this gap, in this paper we reported a new database of underwater swimming pool images for the first time, which is composed of 1500 images and associated subjective ratings recorded by 16 inexperienced observers. In addition, we proposed a main target area extraction and multi-feature fusion image quality assessment (MM-IQA) for a swimming pool environment, which performs pixel-level fusion for multiple features of the image on the premise of highlighting important detection objects. Meanwhile, a variety of well-established full-reference (FR) quality evaluation methods and partial no-reference (NR) quality evaluation algorithms are selected to verify the database we created. Extensive experimental results show that the proposed algorithm is superior to the most advanced image quality models in performance evaluation and the outcomes of subjective and objective quality assessment of most methods involved in the comparison have good correlation and consistency, which further indicating indicates that the establishment of a large-scale pool image quality assessment database is of wide applicability and importance.

## 1. Introduction

The acquisition of underwater images plays a significant role in the research of underwater rescue and biometric tracking at swimming pools in Fei et al. (2012), Alshbatat et al. (2020), and Pleština et al. (2020). However, since the underwater environment is always complicated and variable, this would lead to can result in inaccurate judgments if the unprocessed images extracted from the swimming pool are analyzed directly. Image quality assessment (IQA) has contributed significantly to the study of plentiful many visual signal applications (Wang, 2011), including image transmission, enhancement, and restoration, so the underwater image quality evaluation of swimming pools will open up the possibility for future visual research tasks. Nevertheless, to the best of our knowledge, limited efforts have been made so far to gather a database of swimming pool images and to identify a reliable benchmark for objective quality models.

In recent years, a large number of IQA approaches have been proposed, which mainly contain subjective and objective evaluation methods. Human beings, as the ultimate recipients of visual signals, have the highest voice in judging best ability to judge the quality of images. But subjective assessment methods involving humans are somewhat expensive, time-consuming, and not very useful for practical applications. Therefore, it is urgent necessary to design an objective evaluation method that can simulate the human visual system (HVS) to automatically measure the image quality. So far, these objective IQA approaches can be classified into the following three categories based on the degree of reference to the original image information: full reference (FR) method, reduce reference (RR) method, and no reference (NR) method. In the methods proposed by Gu et al. (2017a), FR IQA method, requires all the information of the original image. After decades of development, it has formed a relatively complete theoretical system and a mature evaluation framework. As the opposite of Unlike the FR method, NR IQA does not require any information of on the original image. Since it is not easy to obtain the original image in some cases, this method has attracted the attention of scholars in recent years (Gu et al., 2015b; Min et al., 2018), and RR method, which is involved in Chen et al. (2021), can obtain some information of the image. This method evaluates the image quality by comparing the difference between the extracted reference image and the partial information of the distorted image.

The most reliable FR IQA methods in the early days are have traditionally been mean square error (MSE) and peak signal-to-noise ratio (PSNR), which are statistical measurements based on image pixels. Although these methods are simple and easy to understand, the results obtained from their evaluation are very different from vary based on the subjective perceived quality of the images. Since then, there are a large number of researchers. There has been significant work carried out toward working on quality assessment models that simulate the human visual system, such as Chandler and Hemami (2007), and so on. One of the most popular algorithms based on HVS is structural similarity (SSIM) presented by Wang et al. (2004), which focuses on extracting the information of brightness, contrast, and structure from reference images. Afterwards, many extensions of the SSIM have been put forward successively. Inspired by the natural scene statistics (NNS) pointed out by Simoncelli and Olshausen (2001), Sheikh et al. resolved the IQA question from the viewpoint of information theory, and they put forward the information fidelity criterion (IFC) mentioned in Sheikh et al. (2005) and its extension version, which is called as the visual information fidelity (VIF) index in Sheikh and Bovik (2006). Zhang et al. (2011) proposed another impactive evaluation algorithm named the feature similarity (FSIM), which selects phase consistency information and gradient information as its two features. Blind parameter algorithm solves the important problem that the original image cannot be obtained. The traditional FR IQA algorithm proposes many gradient evaluation functions from the perspective of image sharpness, such as Brenner gradient function, Tenengrad gradient function, and Laplacian gradient function, etc. Through methods mentioned above can judge the level of image sharpness to a certain extent, there may be major errors for different types of images or scenes. After that, image quality assessment methods based on Natural scene statistics (NSS) emerged. The most typical model is dubbed blind/referenceless image spatial quality evaluator (BRISQUE), an RR IQA method in the spatial domain, which was proposed in Mittal et al. (2012). Other experts and scholars have also made great contributions to this kind of very practical algorithm. Gu et al. (2018) and Gu et al. (2014) have provided corresponding solutions to problems such as huge data and diverse distortion based on the RR IQA model. With the advent of the era of big data, a series of deep learning network structures have shown great advantages in the application of image processing, such as environmental protection (Gu et al., 2020a, 2021b; Liu et al., 2021), PM_2.5_ forecast (Gu et al., 2019, 2021a), and air quality prediction (Gu et al., 2020b). Extensive Considerable attention from researchers has been given to evaluating image quality with deep learning (Hou et al., 2015; Liu et al., 2019) in the past few years. There is no need to define image features as, it relies on a unique deep structure to learn important features of the distorted image so as to predict the image quality score. In recent years, many scholars have improved the IQA methods mentioned above, so there are a large number of IQA methods with high accuracy and stability.

Despite the success of plentiful many IQA methods, there is still a long way to go when it comes to studying a new complex pool environment. To this end, in this paper, we created a large pool database in the first step, and then we proposed the MM-IQA model for the pool environment to objectively evaluate the quality of the database. Finally, we conducted the comparison experiments among available FR IQA and NR IQA methods on the swimming pool image database and, analyzed the advantages and disadvantages of different algorithms;, and the results show that the database is effective and valuable, which and can be used for the future visual research of the pool environment.

The rest of this paper is organized as follows. Section 2 first introduces the swimming pool underwater image dataset. In section 3, we propose an image quality evaluation method based on main object extraction and multi-feature fusion and introduce the quality evaluation method for comparison in experimental parts. Experiments and analysis conducted on our proposed database are reported in section 4. Finally, we conclude our paper in section 5.

## 2. Swimming Pool Image Dataset

Although IQA has made great progress in many areas involving underwater images, very little research has been done in the last decades specifically for the particular scene of swimming pools. In order to make the underwater images of swimming pools more objective to restore the real scene and better reflect the underwater information, so as to meet the actual research needs, we construct a novel and appropriative database of swimming pool images in this paper, which are taken at different shooting angles, locations, and different brightnesses. The process of creating the database will be described at length in the following sections.

### 2.1. Original Image Creation and Filtering

**Figure 1:**
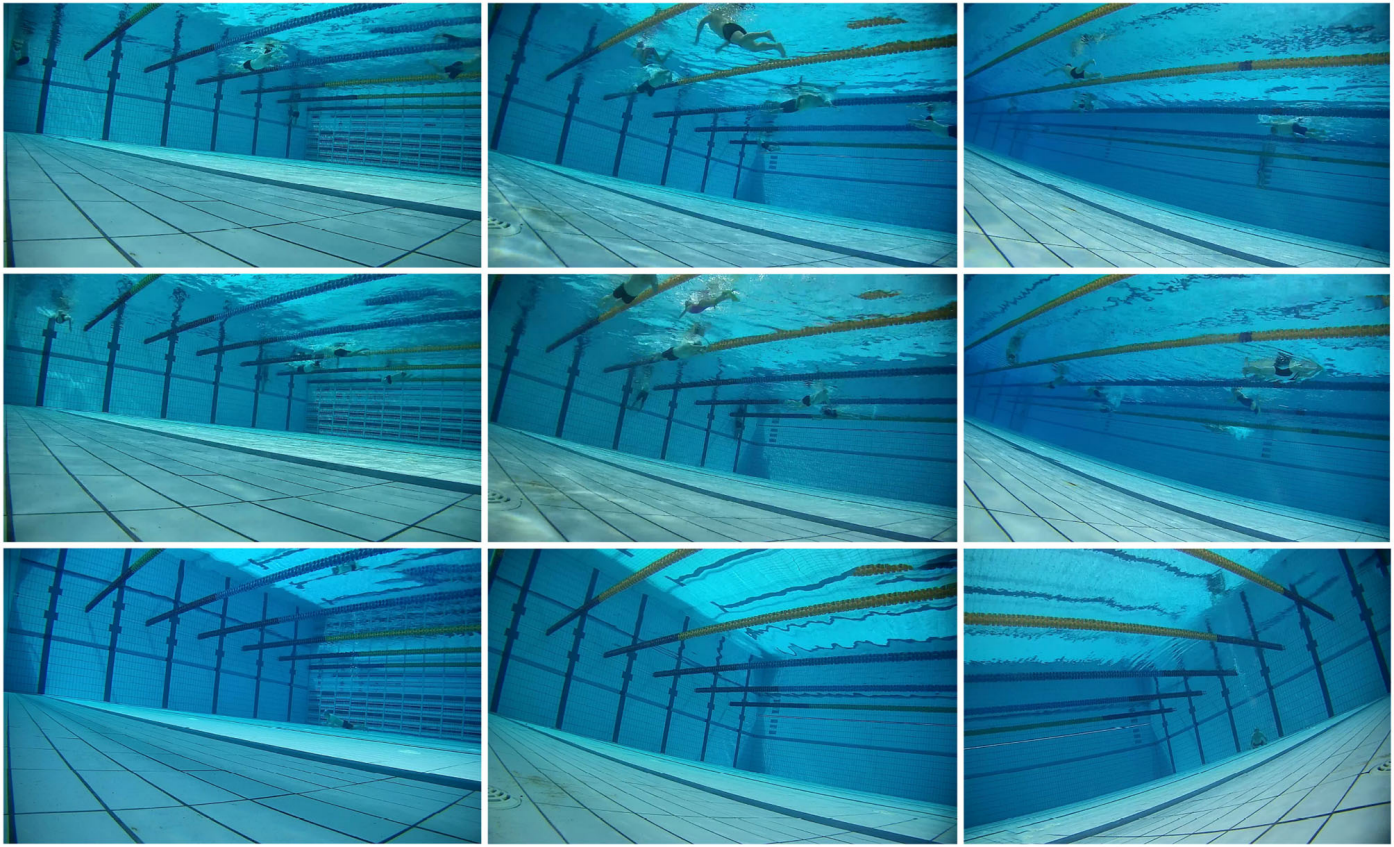
Nine lossless color images in the swimming pool database.

We selected two natatoria for data collection on the spot, one of which is the swimming pool of Ordos Stadium in Inner Mongolia, and the other is the swimming pool of North China University of Science and Technology. We used the same equipment to collect pool images and choose cameras with different angles to acquire images in order to construct a more effective dataset. As the acquisition process is continuous, the similarity of these collected photos is high. Therefore, we filter the 3,000 frames collected when selecting the reference images to obtain more images with different features. In addition, our dataset includes images of simulated drowning pools and pools without people. It is worth noting that, to further ensure the standardization of the IQA database, all reference images are selected according to the uniform size of the original image. To sum up, our presented pool underwater database includes 150 raw images with a resolution of 1920 × 1080, as shown in Figure 1.

### 2.2. Distortion Type and Distortion Level

Digital images often differ from the real environment;, for a particular scene, the distortion type should be judged first. After determining the distortion type, the performance of quality evaluation in subsequent research can be improved (Min et al., 2019a,b). There are many types of image distortion, including blurring, JPEG compression, noise injection, etc. Actually, the damaged image is complex, mainly reflected in many types of distortion, distortion degree, and so on, which requires us to fully consider all possible situations. The, integrated learning method has been proposed accordingly (Gu et al., 2017a). Considering that we are still in the early stages of this research area, we chose only one distortion type to process the database. The type of distortion chosen here is JPEG compression, which is a common lossy compression format for images. The compression process can be divided into five steps: image segmentation, color space transformation, discrete cosine transformation, data quantization, and coding.

**Figure 2:**
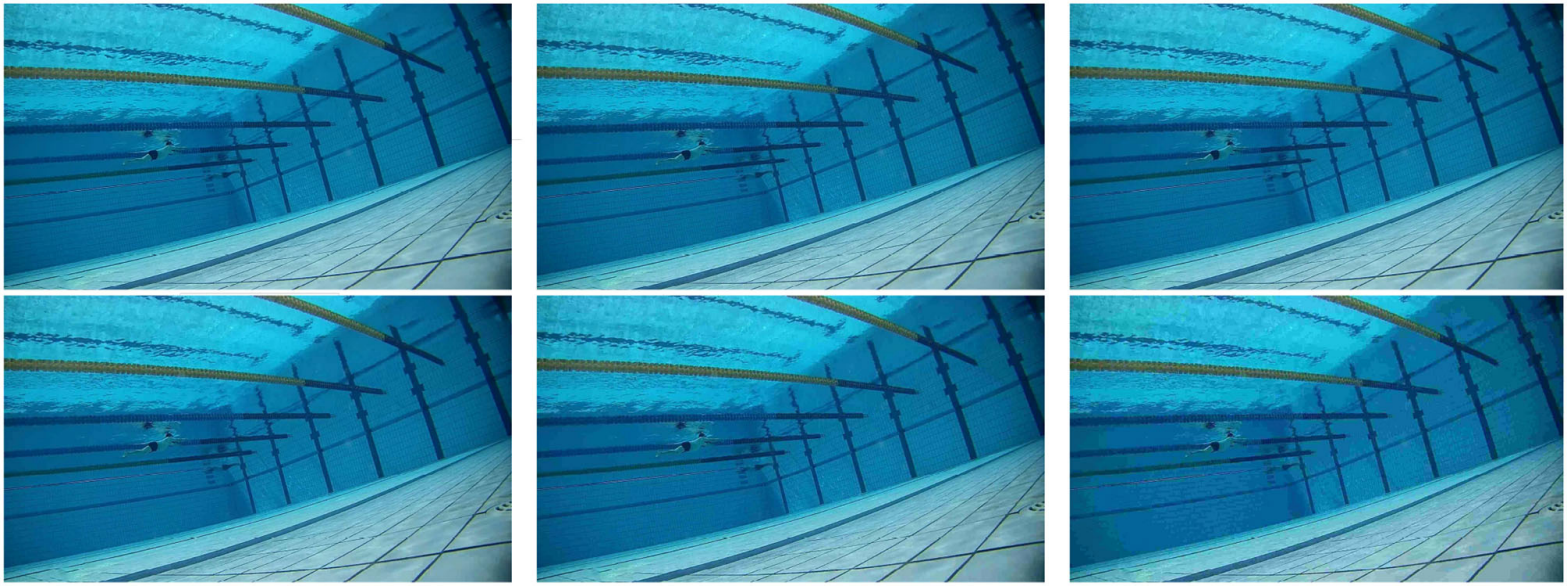
One original image and its five distorted images vary from 10 to 50.

We use the inwriter command in Matlab to generate JPEG compressed images, by setting the parameter *Q*, we can get images with compression levels of 10, 20, 30, 40, and 50 (distortion), as shown in Figure 2. In this way, we have a quality evaluations database in swimming pools.

### 2.3. Subjective Evaluation Process

In fact, when people evaluate the quality of an image, many factors should be taken into consideration, including not only the factors of the image itself, but also the psychological factors of subjects and the external environment. The distance between the observer and the image is studied in Gu et al. (2015a). According to ITU-R BT.500-1 in Union (2002), our subjective viewing test experiment is conducted with a single-stimulus method. In this process, we select 16 inexperienced subjects, most of whom are college students from various professional fields. The an interactive system is designed by using MATLAB, so as to automatically display the images and collect the original subjective scores, which are represented by $x_{ab}^{\prime }$. To reduce the influence of memory on opinion scores, the presentation order is provided randomly to the observers who are asked to give their overall sensation of quality on a continuous quality scale of 1 to 5. Table 1 summarizes many critical parameters of the subjective testing environment.

**Table 1:** Subjective experimental conditions and parameters.

**Method**	**Single-stimulus(ss)**
Evaluation scales	Continuous quality scale from 1 to 5
Color depth	24
Image coder	Joint Picture Group(JPG)
Subject	Sixteen inexperienced subjects
Image resolution	1,920 × 1,080
Viewing distance	Four times the image height
Room illuminance	Dark

We calculate all of the gathered differential mean opinion scores (DMOS) after the viewing test experiment. Here, we denote the subjective assessment score on the distorted image *I*_*b*_ as *a* and the number of distorted images as *b*, where *a* = {1, …, 16} and *b* = {1, …, 1500}. In addition, we set *x*_*ab*_ to indicate the score of the primitive images. Then, the following steps are shown below:

• Outliers screening: Due to the large number of test pictures, it is impossible for subjects to maintain a high level of attention at all times, which can lead to outliers. To solve this issue, we adopt the method proposed by Ponomarenko et al. (2009) to screen the outliers of the scores. Specifically, we treat this value with caution when the original DOMS value of an image is outsides the standard deviation of the mean score of this image.

• Differential scores: Subtracting the score of original images from its reference image, which can be expressed as $D_{ab}=x_{ab}-x_{ab}^{\prime }$.

• Average score: The DMOS value for the image is defined as $\frac{1}{N_{A}}\underset{a}{\sum }D_{ab}$, where *N*_*A*_ is the number of subjects.

## 3. Methodology

The objective evaluation method of image quality, which realizes the accurate and automatic perception of image quality through specific formulas, replaces the subjective visual system of human eyes. In the past decades, a large number of evaluation criterions have been put forward to assess the quality of images. In this section, we will start with a detailed introduction of the MM-IQA algorithm, followed by an overview of some classic quality evaluation algorithms involved in comparison.

Higher recognition speed is desirable for underwater visual research in swimming pools, especially when it involves underwater tasks such as target recognition and tracking and rescue assistance, which often requires high speeds of recognition. Therefore, we put forward an image quality evaluation method based on main target area extraction and multi-feature fusion for swimming pool images. To begin with, because the sensitivity of vision to distortion varies in different areas, the main target area is separated from the large- scale reference image and distortion image of the swimming pool image. Then, the brightness, contrast, and gradient information extracted from the small-scale image are fused into local structure information. Finally, we obtained the image quality evaluation results by structural fusion of the two scales.

### 3.1. Main Target Extraction

It is known to all accepted that the information of the outside world is huge while the processing capacity of the human sensory nervous system is limited. Human visual processing can be naturally divided into two stages: the self-processing process of distributed attention, parallel processing, and automatic feature registration;, and then, the controlled processing process of attention concentration and feature integration. Zhang et al. (2020), Tang et al. (2020), and Emoto (2019) noted, based on their observations, that the HVS tends to focus on interesting areas of the images when viewing and judging the quality of each distorted image. Furthermore, numerous studies have shown that in computer vision tasks, the method of dividing the target region into the main region first and then studying the main region can greatly accelerate the detection speed. In the paper, different degrees of distortion do not affect the location of prominent targets (pool wall, swimmer, drowning person) in the pool. Therefore, we believe that the main target area extraction can be used as a contributor to improve the performance of the pool environmental quality assessment algorithm.

The last decade has witnessed the development and expansion of the extraction of the main target region, which has been applied in various researches studies, e.g., Image quality evaluation (Gu et al., 2016a), target tracking (Gongguo et al., 2020), and target recognition (Gu et al., 2021b). In 2007, Hou and Zhang (2007) proposed a significance detection method based on spectral residuals. After a series of operations including number spectrum analysis, spectral residuals extraction, and spatial domain mapping of the input image, the region where the main target is located was finally obtained. Fast Fourier Transform (FFT) and Inverse Fast Fourier Transform (IFFT) are known to us for the characteristics of fast detection speed and high frequency information accessibility. And the improved versions of this method these methods are used in our model for extracting the main target contour of the image. Before processing the image in frequency domain, we transformed the pixel coordinates of two-dimensional images of the spatial domain into the spectral coordinates of the frequency domain by using Fourier transform. Hence, the FFT of image *f*(*a, b*) can be defined as:


(1) $$\begin{array}{l} \begin{array}{c} F\left(\mu ,\theta \right)=\frac{1}{PQ}\sum_{a=0}^{P-1}\sum_{b=0}^{Q-1}f\left(a,b\right)e^{-j2\pi \left(\frac{\mu a}{P}+\frac{\mu b}{Q}\right)} \end{array} \end{array}$$

where *P*, *Q* represent the size information of the image, *a* and *b* are the spatial variables of the image, and μ and θ are the frequency variables of the image.

The spectrum of the image *h*(*x*) is divided into amplitude spectrum A(f) and phase spectrum P(f). In order to suppress the influence of noise in the process of image acquisition, we stretched the amplitude spectrum to get keep the energy of different pixel values in a small gap interval. Then, We normalized the stretched $A^{\prime }\left(f\right)$ to get $\bar{A}\left(f\right)=\frac{\sum A\left(f\right)}{\sum A^{\prime }\left(f\right)}A^{\prime }\left(f\right)$, the spectral residual R(f) can be computed by subtracting the product of $\bar{A}\left(f\right)$ and δ from $\bar{A}\left(f\right)$. By using IFFT, the main target region map is constructed in the spatial domain. The values of each pixel in the primary target area are then squared to indicate the estimation error. Finally, smooth the saliency map was smoothed with a Gaussian filter *g*(*x*) to achieve a better visual effect. The whole process is as follows:


(2) $$\begin{array}{l} \begin{array}{c} A\left(f\right)=\log\left(\left|F\left[h\left(x\right)\right]\right|\right),\\ P\left(f\right)=\varphi \left(F\left[h\left(x\right)\right]\right),\\ A\prime \left(f\right)=A^{\gamma }\left(f\right),\\ R\left(f\right)=\bar{A}\left(f\right)-\delta \bar{A}\left(f\right),\\ P_{mt}=g\left(x\right)\cdot F^{-1}{\left[e^{R\left(f\right)+P\left(f\right)}\right]}^{2} \end{array} \end{array}$$

where F and $F^{-1}$ represent the FFT operator and the IFFT operator, δ is the 7 × 7 identity matrix for mean filtering.

Considering that the difference of the main target area is mainly reflected in the target contour, we further extract the contour information. We select the similarity between the reference image and the distorted image as the contour information, which is a simple and effective method.


(3) $$\begin{array}{l} \begin{array}{c} Con\left(x,y\right)=\frac{2P_{Mt_{x}}\cdot P_{Mt_{y}}+C1}{P_{Mt_{x}}^{2}+P_{Mt_{x}}^{2}+C1} \end{array} \end{array}$$

where the constants C1 is set to increase the stability when the denominator is close to zero.

In addition, we found that different areas of the pool contributed differently to the quality of the human perceived image. For example, it is easier to draw conclusions by observing the tiles on the pool walls and the swimmers when the distortion is low. Therefore, location information is also essential for similarity evaluation. We use *P*_*M*_*t*__*w*__ = (*Mt*_*x*_ · *g*(*x*)) ∪ (*Mt*_*y*_ · *g*(*x*)) to weight the global similarity;, *g*(*x*) is a gaussian matrix whose function is to eliminate noise. After adding location information, we can get the final global structure *G*_*s*_ :


(4) $$\begin{array}{l} \begin{array}{c} G_{s}=\frac{\sum_{\Omega }Con{\left(x,y\right)}^{\psi }\cdot P_{Mt_{w}}\left(x,y\right)}{\sum_{\Omega }P_{Mt_{w}}\left(x,y\right)} \end{array} \end{array}$$

where Ω are the whole spatial domain, and parameter ψ is used to adjust the relative importance of global structure.

### 3.2. Multi-Feature Fusion

The pool environment is complex and easily affected by the external environment. Generally speaking, the fusion of a variety of information can make up for the deficiency, which will make the experimental results more complete and convincing (Gu et al., 2020b, 2021a). So, in order to better describe the distortion degree of the pool image, we compare the reference image with the distorted image from local brightness, local contrast, and local clarity. The characteristic of vision is non-linear, it being too bright or too dark will cause varying degrees of damage to the quality of the image. As the bottom feature of image, brightness feature will directly affect the result of image quality evaluation (Mantel et al., 2016). The basic information of the image or pixel can be obtained from the brightness characteristics. When the brightness value is lower than a certain value, the details of an image will become difficult to observe, and the image quality will also deteriorate if the image is overexposed. The average intensities of reference image *x* and distorted image *y* are calculated, respectively:


(5) $$\begin{array}{l} \begin{array}{c} \mu_{x}=\frac{1}{N}\sum_{i=0}^{N}x_{i},\mu_{y}=\frac{1}{N}\sum_{i=0}^{N}y_{i} \end{array} \end{array}$$

where μ_*x*_ and μ_*y*_ represent the local brightness of reference and distorted pool images, respectively. And then, for luminance comparison, the similarity measurement method has been used between μ_*x*_ and μ_*y*_:


(6) $$\begin{array}{l} \begin{array}{c} P_{l\left(x,y\right)}=\frac{2\mu_{x}\cdot \mu_{y}+C2}{\mu_{x}^{2}+\mu_{y}^{2}+C2} \end{array} \end{array}$$

where the constants C2 has the same function as C1.

As the key to the visual effect, contrast reflects the sharpness of the image and the depth of the grooves in the texture. Generally speaking, high contrast is of great help to image clarity, detail performance, and gray level performance. On the contrary, a low image contrast usually causes the whole image to be blurred. Signal contrast is mainly obtained by estimating the standard deviation (square root of variance) of the image, and the standard deviation of discrete signal is calculated as:


(7) $$\begin{array}{l} \begin{array}{c} \sigma_{x}={\left[\frac{1}{N-1}\sum_{i=0}^{N}\left(x_{i}-\mu_{x}\right)\right]}^{\frac{1}{2}},\\ \sigma_{y}={\left[\frac{1}{N-1}\sum_{i=0}^{N}\left(x_{i}-\mu_{y}\right)\right]}^{\frac{1}{2}} \end{array} \end{array}$$

where σ_*x*_ and σ_*y*_ represent the local brightness of reference and distorted pool images, respectively. Similarly, for contrast comparison, the similarity measurement method has also been used between σ_*x*_ and σ_*y*_:


(8) $$\begin{array}{l} \begin{array}{c} P_{c\left(x,y\right)}=\frac{2\sigma_{x}\cdot \sigma_{y}+C3}{\sigma_{x}^{2}+\sigma_{y}^{2}+C3} \end{array} \end{array}$$

where the constant is C3 has the same function as C1 and C2.

Besides contrast and brightness, sharpness feature is another important image feature, which includes sharpness of image plane and sharpness of image edge. More attention has been paid to the edge of the image when it comes to sharpness feature (Tao et al., 2014; Sheng et al., 2015), which also makes up for the lack of contrast sensitivity in this aspect of contrast. Image edge is a set of pixels connected by the boundary between two regions of an image. We can use gradient feature to fully describe the information of image edge structure and contrast change. Commonly used operators for calculating gradients include the Sobel operator, the Prewitt operator, and the Scharr operator. Here, we used the Scharr gradient operator to extract gradient information of reference image *x* and distorted image *y*, respectively:


(9) $$\begin{array}{l} \begin{array}{c} S_{h}=\left[\begin{array}{ccc} 3 & 0 & -3\\ 10 & 0 & -10\\ 3 & 0 & -3 \end{array}\right]\times \frac{1}{16},S_{v}=\left[\begin{array}{ccc} 3 & -10 & 3\\ 0 & 0 & 0\\ -3 & -10 & -3 \end{array}\right]\times \frac{1}{16} \end{array} \end{array}$$

where *S*_*h*_ and *S*_*v*_ are separately represent the Scharr convolution masks along the horizontal and vertical directions, which are used for gradient extraction of the image. We can obtained the gradient magnitudes of *x* and *y*, denoted as *s*_*x*_ and *s*_*y*_, which are given by:


(10) $$\begin{array}{l} \begin{array}{c} s_{x}=\sqrt{{\left(S_{h}*x\right)}^{2}+{\left(S_{v}*x\right)}^{2}},s_{y}=\sqrt{{\left(S_{h}*y\right)}^{2}+{\left(S_{v}*y\right)}^{2}} \end{array} \end{array}$$

where symbol “*″ indicates the convolution operation. Then the difference between *s*_*x*_ and *s*_*y*_ can be written as:


(11) $$\begin{array}{l} \begin{array}{c} P_{s\left(x,y\right)}=\frac{2s_{x}\cdot s_{y}+C4}{s_{x}^{2}+s_{y}^{2}+C4} \end{array} \end{array}$$

where the constant is C4 has the same function as C1, C2, and C3.

By structure-fusion of the three local features of brightness, contrast, and sharpness in the small-scale range with the main target region extraction in the large-scale range, we obtained the final MM-IQA metric:


(12) $$\begin{array}{l} \begin{array}{c} MM-IQA=\frac{\sum_{\Omega }{\left[P_{s}+w_{1}P_{l}\cdot P_{c}\right]}^{\theta }Con{\left(x,y\right)}^{\psi }\cdot P_{Mt_{w}}}{\sum_{\Omega }P_{Mt_{w}}} \end{array} \end{array}$$

where *P*_*s*_ + *w*_1_*P*_*l*_ · *P*_*c*_ presents a fusion of three local features, *w*_1_ is a weight parameter, and θ has the same function as ψ.

## 4. Experimental Results and Analysis

### 4.1. Performance Measures

This section will conduct a wide range of experiments on our constructed database to assess the accuracy of these methods mentioned above. The swimming pool image database is a large-scale IQA database with 1500 images generated from 150 pristine images, having 5 five distortion levels and 1 one distortion type, therefore it is chosen as the testing bed. As per the suggestion given by Corriveau (2017), we first map the prediction outputs of each IQA metrics to subjective scores using non-linear regression with the five-parameter logistic function, which is regarded as:


(13) $$\begin{array}{l} \begin{array}{c} S\left(q\right)=\tau_{1}\left\{\frac{1}{2}-\frac{1}{1+e^{\left(q-\tau_{3}\right)\tau_{2}}}\right\}+q\tau_{4}+\tau_{5} \end{array} \end{array}$$

where *q* and *S*(*q*) are the input and mapped scores, and the regression model parameters τ_1_ to τ_5_ are to be determined during the curve fitting process.

Then, we evaluate the IQA index using five commonly used performance indicators, where the Spearman rank order correlation coefficient (SROCC) and the Kendall rank order correlation coefficient (KROCC) are applied for evaluating to evaluate the monotonicity of prediction. The third index is Pearson linear correlation coefficient (PLCC), which estimates the prediction accuracy by measuring the correlation between the MOS and objective fractions after non-linear regression. Finally, in order to evaluate the prediction consistency, we also use the Root mean square error (RMSE) and the Mean absolute error (MSE) between S(q) and q.

### 4.2. Methods for Comparison

In this paper, we used the classical and the latest FR IQA method and part of NR IQA method to conduct a comparative experiment with MM-IQA in the underwater database of swimming pools. The methods involved in the experiment are shown below:

• The MSE, PSNR, and SSIM proposed by Wang et al. (2004), are the benchmark IQA methods that are widely used in image processing researches.

• NQM in Damera-Venkata et al. (2000), quantifies the effects of linear frequency distortion and noise injection on HVS.

• FSIM and FSIMc from Zhang et al. (2011), apply phase congruency and gradient magnitude to represent the local quality of the image based on the fact that the HVS understands images mainly from the low-level features of the images.

• IGM in Wu et al. (2013), who decomposes the reference image into a predicted part and a disordered part according to the Bayesian prediction model. In addition, the PSNR and SSIM values are used to measure the noise energy of these two parts, respectively. Finally, we combine the two results to obtain the overall mass score.

• MS-SSIM pointed out by Wang et al. (2003), performs the SSIM in different scales and integrates their outputs with psychophysical weights.

• VIF and VIFP, quantify the Shannon information shared between the reference and distorted images in Sheikh and Bovik (2006) by using a unified information fidelity criterion based on NSS, distortion, and HVS modeling.

• MAD presented by Chandler (2010), combines two different strategies based on detection and appearance. When the quality of the image is high, local brightness and contrast masking can be used to estimate the perceptual distortion based on detection, while variations in local statistics of spatial frequency components are used to estimate appearance-based perception distortion in low-quality images.

• GSI developed by Liu et al. (2012), emphasizes on the similarity of gradient sizes plays which play an important role in scene understanding.

• GMSD is designed by Xue et al. (2014), and predicts visual quality score by using the standard deviation of the similarity graph of the gradient amplitude between the reference image and the distorted image, which meets both the time and efficiency requirements.

• VSI presented by Zhang et al. (2014), which would integrate visual saliency into IQA metrics.

• ADD1 and ADD2 in Gu et al. (2016b), new aggregation models in IQA, which proposed via analyzing the distortion distribution of image content and distortion effects.

• PSIM from Gu et al. (2017a), combines two scales of the GM similarities, both of which are color information similarity, and a reliable perceptual-based pooling, respectively.

• BRISQUE in Mittal et al. (2012), an NR IQA method based on natural scene statistics who that uses scene statistics of local normalized luminance coefficient to quantify distortion.

• NIQE pointed out by Mittal et al. (2013), is proved to be a simple and efficient quality assessment algorithm who that calculates the deviation only and only relies on the statistical rules in natural images without training the artificially assessed distorted images.

• SISBLIM proposed by Gu et al. (2014), takes the multi-distortion image problem as the research object and evaluates image quality from six parts: noise estimation, image deionizing, blur measure, JPEG-quality evaluator, joint effects' prediction, and HVS-based fusion.

• NIQMC from Gu et al. (2017b), an NR IQA based on the concept of information maximization who that considers both local and global information to generate the quality fraction of the contrast distortion image.

• ASIQE presented in Gu et al. (2017c), which quantifies the effects of image complexity, screen content statistics, overall brightness quality and detail sharpness on HVS, is commonly used to evaluate the quality of screen content images.

### 4.3. Overall Performance Evaluation

In order to better verify the effect of objective IQA method and subjective consistency, we test and calculate the objective IQA algorithm on a subjective IQA database. Tables 2, 3 illustrate the performance results of PLCC, SROCC, KROCC, RMSE, MSE of FR IQA, and NR IQA on the new pool database, respectively. At the bottom of this these two tables is the performance of MM-IQA method shown in bold, and the best models for both FR IQA and NR IQA algorithms used for comparison are also shown in bold.

**Table 2:** Performance comparison of FR-IQA metrics on the pool image database.

**Metrics**	**SROCC**	**KROOC**	**PLCC**	**MSE**	**RMSE**
MSE	0.8659	0.6591	0.4662	0.3616	0.4773
PSNR	0.8695	0.6591	0.4662	0.3537	0.4714
SSIM	0.8779	0.6940	0.5064	0.3416	0.4570
NQM	0.8546	0.6379	0.4527	0.3748	0.4955
VIF	0.8817	0.6931	0.5054	0.3380	0.4502
IGM	0.8842	0.6888	0.5014	0.3337	0.4457
FSIM	0.8835	0.6918	0.5037	0.3376	0.4469
FSIMc	0.8834	0.6885	0.5004	0.3376	0.4472
**MS-SSIM**	**0.8859**	**0.6976**	**0.5097**	**0.3321**	**0.4426**
MAD	0.8740	0.6734	0.4842	0.3489	0.4638
GSI	0.8820	0.6830	0.4942	0.3389	0.4497
GMSM	0.8840	0.6819	0.4948	0.3348	0.4461
GMSD	0.8833	0.6749	0.4859	0.3359	0.4474
PAMSE	0.8802	0.6740	0.4879	0.3394	0.4529
VSI	0.8799	0.6954	0.5107	0.3400	0.4534
SWGSSIM	0.8829	0.6769	0.4869	0.3375	0.4481
ADD1	0.8859	0.6944	0.5077	0.3327	0.4427
ADD2	0.8838	0.6753	0.4876	0.3358	0.4465
PSIM	0.8838	0.7197	0.5314	0.3348	0.4465
**MM-IQA**	**0.8934**	**0.7508**	**0.5675**	**0.3246**	**0.4287**

**Table 3:** Performance comparison of RR-IQA metrics on the pool image database.

**Metrics**	**SROCC**	**KROOC**	**PLCC**	**MSE**	**RMSE**
BRISQUE	0.6540	0.5392	0.3783	0.5529	0.7219
**SISBLIM-SM**	**0.8901**	**0.7535**	**0.5734**	**0.3343**	**0.4348**
SISBLIM-WM	0.8861	0.7384	0.5560	0.3341	0.4432
NIQE	0.8787	0.7549	0.5702	0.3559	0.4555
ASIQE	0.8630	0.6851	0.5013	0.3612	0.4821
**MM-IQA**	**0.8934**	**0.7508**	**0.5675**	**0.3246**	**0.4287**

The performance of the same quality evaluation algorithm varies from different databases. For the FSIM algorithm, the result of SROCC in the swimming pool image database is 0.8835, while the SROCC result of the same algorithm in the LIVE database is 0.9634, which is pointed out by Sheikh (2003). In addition, due to the good correlation between subjective score and objective evaluation results, our proposed database can also be used to compare the performance of some IQA algorithms, e.g., the extended algorithms MSSSIM obtains better performance than SSIM. We can transform the pool images into grayscale for further study in that pool images are always single singular in color. In this regard, we can conclude from the results that FSIM using gray scale images achieves better results than FSIMc. Surprisingly, the non-parametric algorithms also perform the task of visual evaluation better on the pool database, and even some of the non-parametric algorithms perform better than the mature parametric algorithms. In terms of the overall experimental results, the large-scale IQA database created in this paper shows good consistency in testing different IQA algorithms, which also proves the effectiveness of the database.

## 5. Discussion and Conclusion

As an interactive form of information, images are playing an increasingly important role in the field of multimedia. Yet the amount or importance of the information conveyed by images is not only related to the content and the format of images, but also to the image quality. In general, the higher the quality of the image, the more information people can receive and perceive by looking at the image. In At present, IQA method is becoming more and more important in the field of image processing and computer vision, and is widely used in different practical scenarios.

As a new research field, the swimming pool image research has also been more and more people's attention been gathering increasing attention in recent years, at present there are a lot of swimming pool water to carry on many areas in which to ask research questions, such as swimming pool environment anomaly detection, swimming pool body posture recognition, swimming pool, target tracking, etc., and the image quality is the basis of all vision problems, so the establishment of the swimming pool image database is very necessary. After establishing the database, we evaluated the subjective and objective image quality, respectively, then used three correlation indices, SROCC, KROCC, and PLCC, to describe the consistency between the subjective IQA approach and the objective IQA method, and finally measured the error of the objective image quality score with MOS by using MSE and RMSE. The results of the experiment show that the subjective and objective evaluation can match well, but as the swimming pool environment is easily disturbed by the external environment (such as light, shade, and water ripples). In the future, we will select more distortion types to process the images in our database and further consider the characteristics of the swimming pool environment, so as to seek a more appropriate IQA model and make contributions to the practical research.

## Data Availability Statement

The raw data supporting the conclusions of this article will be made available by the authors, without undue reservation.

## Author Contributions

FL conceived the framework of the paper and implementation and wrote the manuscript. SL assisted in algorithm conception and interpretation of the results. SX participated in the revision and content supplement of the article. JL revised the layout of the article and checked for grammatical errors.

## Conflict of Interest

The authors declare that the research was conducted in the absence of any commercial or financial relationships that could be construed as a potential conflict of interest.

## Publisher's Note

All claims expressed in this article are solely those of the authors and do not necessarily represent those of their affiliated organizations, or those of the publisher, the editors and the reviewers. Any product that may be evaluated in this article, or claim that may be made by its manufacturer, is not guaranteed or endorsed by the publisher.
